# Recurrent Kounis Syndrome: A Case Report and Literature Review

**DOI:** 10.3390/jcm13061647

**Published:** 2024-03-13

**Authors:** Raffaele Brancaccio, Laura Bonzano, Alessia Cocconcelli, Rostyslav Boyko, Giuseppe Ienopoli, Alberico Motolese

**Affiliations:** 1 Dermatology Unit, Azienda Unità Sanitaria Locale-IRCCS di Reggio Emilia, 42122 Reggio nell’Emilia, Italy; laura.bonzano@ausl.re.it (L.B.); alessia.cocconcelli@ausl.re.it (A.C.); alberico.motolese@ausl.re.it (A.M.); 2 Postgraduate School of Allergy and Clinical Immunology, University of Modena and Reggio Emilia, 41125 Modena, Italy; rostyslavboyko@yahoo.it (R.B.); giuseppeienopoli10@gmail.com (G.I.)

**Keywords:** Kounis syndrome, recurrent Kounis syndrome, omalizumab

## Abstract

Kounis syndrome is a condition where inflammatory cells (mostly mast cells with the contribution of macrophages and T-lymphocytes) cause an acute coronary syndrome. Kounis syndrome comes in four variants: type I in patients with normal coronary arteries; type II in patients with inactive pre-existing atheromatous disease; type III in patients with pre-existing coronary artery stenting; type IV in patients with a pre-existing coronary artery bypass. Recently, we came across a case of recurrent type I Kounis syndrome in our clinical practice. The purpose of the paper is to present our case and conduct a review using the Pubmed scientific database about the most relevant cases of recurrent Kounis syndrome. This review shows that recurrent Kounis syndrome is a rare condition and is mostly associated with Kounis syndrome type III. Recurrent Kounis syndrome may be also triggered by vaccination and it could be associated with chronic spontaneous urticaria. In the last condition, therapy is represented by second-generation anti-histamines and corticosteroids, but also by an anti-IgE monoclonal antibody (omalizumab) in the recalcitrant cases.

## 1. Introduction

Kounis syndrome is a cardiovascular disease defined as the concurrence of acute coronary syndrome and coronary spasm, acute myocardial infarction and stent thrombosis. Mast cell degranulation and platelet activation, leading to the coronary spasm due to the liberation of various inflammatory mediators, seem to be involved in the pathogenesis of this condition [1]. Recent studies have shown that Kounis-like syndromes can also affect the mesenteric [2] and cerebral arteries [3]. Kounis syndrome can be induced by different causes, whose number has been increasing in the last years. The most common triggers are antibiotics, non-steroidal anti-inflammatory drugs (NSAIDs), and hymenoptera venom, although other drugs, food, latex, environmental exposure and several health conditions have also been reported as precipitating events [4]. The incidence of Kounis syndrome has been estimated in a retrospective study during a 3-year period [5]. In this study, 226 individuals developed 246 episodes of severe anaphylaxis, which included cardiovascular symptoms, with an incidence of 7.9–9.6 per 100,000 inhabitants per year. Death due to anaphylaxis occurred in three subjects, making the mortality rate 0.0001%. As cases of Kounis syndrome increase, recent reports have shown that this disease has been observed in every ethnic group, age group (from 2 to 90 years old) and geographic location. Kounis syndrome seems not to be a rare disease. However, it is infrequently reported in the literature and diagnosed in clinical practice, essentially due to missed, unrecognized or non-diagnosed cases. Furthermore, there is a lack of trials aiming to determine its exact prevalence and incidence. The highest number of diagnoses come from Southern Europe, especially from Greece, Italy and Spain. This geographical “predilection” could be attributed to different causes: increased awareness of physicians, climate and environmental conditions (e.g., pollen allergy cross-reactivity, exposure to hymenoptera venom), medication overuse, failure of preventive measures, etc. Mast cells play a pivotal role in the development of the pathogenesis of the disease. Mast cells interact via multidirectional stimuli with other phlogistic cells. These components include platelet-activating factor (PAF), histamine, arachidonic acid metabolites, proteases and a large number of cytokines and chemokines. All these phlogistic mediators are produced and released during the allergic activation process. Other inflammatory cells such as macrophages and T-lymphocytes are involved [1,6]. Platelet activation plays a key role in the development of the clinical manifestations of the syndrome. Thrombocytes are activated by the binding of the ligand to one of the receptors (FcγRI, FcγRII, FcεRI, FcεRII) located on their surface [7]. The final result is a self-reinforcing multidirectional vicious circle of activation of different phlogistic pathways and various cytokine cascades [1,8]. Many different inflammatory mediators are produced by mast cells and stored in approximately 500 secretory granules per cell. Furthermore, many inflammatory mediators are synthesized de novo, enriching the pool of those already preformed. These components are released locally and into the bloodstream when a specific antigen binds to the IgE antibodies attached to the surface of the mast cells and induces massive degranulation. Mast cells have been found in different human organs and systems including the heart and the coronary arteries [9]. The initiation of an allergic reaction takes place when allergens (ligands) cause a cross-bridge of IgE-specific antibodies (receptors) on the mast cell or basophil cell surface [10]. A few studies have also reported Kounis syndrome without obvious allergic symptoms, which is why its etiology remains unclear. Various newly formed and accumulated inflammatory mediators are released from mast cells: biogenic amines such as histamine and cytokines; enzymes such as neutral protease chymase, tryptase and cathepsin D; peptides; proteoglycans; cytokines; growth factors; and arachidonic acid products such as leukotrienes, thromboxane, prostacyclin, PAP and tumor necrosis factor. Histamine causes coronary vasoconstriction and induces tissue factor expression and platelet activation. Proteases activate matrix metalloproteinases, which degrade the collagen envelope and cause plaque erosion and rupture, while tryptase possesses fibrinolytic properties [1]. Furthermore, chymase and cathepsin D stimulate angiotensin I conversion to angiotensin II, which is a significant vasoconstrictor [11]. Leukotrienes are also vasoconstrictors [12]. Thromboxane is a strong booster of thrombotic aggregation with vasoconstrictor properties [13]. The main clinical signs and symptoms of Kounis syndrome are always accompanied by subclinical, clinical, acute or chronic allergic reactions with cardiac symptoms. Cardiac symptoms and signs are consistently associated with various ECG changes, from ST segment elevation or depression to all types of cardiac arrest and arrhythmias. Four variants of Kounis syndrome have been described in the literature. Kounis syndrome type I affects patients with normal coronary arteries in whom the release of inflammatory mediators induces coronary artery vasospasm with or without raised troponins, which could move forward to myocardial infarction. Type II affects patients with previously inactive atherosclerotic disease whose allergy causes plaque erosion resulting in coronary vasospasm or acute myocardial infarction. Phenotype III involves coronary stent thrombosis caused by an allergic reaction [1]. Type IV is associated with coronary artery bypass graft thrombosis [4]. The diagnosis of Kounis syndrome is based on clinical signs and symptoms, laboratory tests, electrocardiogram, echocardiography and angiography. Recently, modern methods such as heart MRI and myocardial scintigraphy have helped to confirm diagnosis. The measurements of tryptase and cardiac enzymes such as troponins in serum are particularly helpful. The only source of tryptase is mast cells, but the amount of tryptase in the human body is negligible [1]. Like other inflammatory mediators, tryptase is short-lived, with a half-life of approximately 90 min [14]. The optimal time to collect the first sample appears to be 30 min after the onset of symptoms and 30 min within the next 2 h [15]. Cardiac enzymes such as CK, particularly CK-MB, are useful in the diagnosis of cardiac damage associated with allergic or anaphylactic attacks. Echocardiography and coronary angiography are necessary to diagnose cardiac wall abnormalities and to demonstrate the coronary anatomy of Kounis syndrome [1]. Recently, new techniques have been used, such as thallium-201 single-photon emission computed tomography (SPECT) and 1251-15-(p-iodophenium)-3-(R,S)-methylpentadecanoic acid (BMIPP) SPECT have been already used in Kounis syndrome type I and revealed severe myocardial ischemia, whereas coronary angiography showed normal coronary arteries [16]. Dynamic cardiac magnetic resonance (MRI) is also a reliable tool for evaluating cardiac involvement in Kounis syndrome. Late contrast imaging shows normal outflow around a subendocardial lesion in a patient with Kounis syndrome type I [17]. Usually, Kounis syndrome is an isolated phenomenon; nevertheless, a few cases of recurrent Kounis syndrome are reported in the literature. Recently, we came across a case of recurrent Kounis syndrome in our clinical practice. The aim of this paper is to present our case and to collect and review data from the current literature about cases of recurrent Kounis syndrome. The bibliographic search was conducted using Pubmed. The keywords selected for our search process were “Kounis syndrome” combined with “recurrent”. In our review, we included all the research articles indexed in peer-reviewed scientific journals that report cases of recurrent Kounis syndrome, with a total of six articles from six different sources [4,18,19,20,21,22].

## 2. Relevant Sections

### 2.1. Case Report

We report a case of a 47-year-old woman with no cardiovascular risk factors, who was admitted to our Emergency Department due to chest pain. Electrocardiogram showed ST segment elevation and elevated troponin-I levels (3227.6 ng/L) were detected whereas no signs of atherosclerosis were found by coronary angiography. After a few hours after the onset of cardiac symptoms, the patient presented a generalized eruption of widespread wheals and oedema of lips and eyelids. Therefore, oral prednisone and cetirizine treatment was started with rapid regression of both cardiac and skin manifestations. Serum tryptase value was in a normal range (6.2 µg/L), a potential culprit for an IgE-mediated reaction was missing in the patient’s anamnesis and also serum-specific IgE for food allergens was negative. Considering that also dyspnea, vomiting, diarrhea, hypotension and syncope were lacking and that epinephrine was not administered because corticosteroids were able to control the symptoms, the option of anaphylaxis was ruled out. Therefore, we considered urticaria-angioedema associated with type I Kounis syndrome to be the most suitable diagnosis. Furthermore, anamnesis revealed that the patient was vaccinated for SARS-CoV-2 (BNT162b2) six days before the symptom occurrence and that, 15 days before vaccination, a skin prick test and an intra-dermoreaction test with polyethylene glycol (PM 4000) were performed showing no hypersensitivity to this substance. The patient was discharged from hospital after 17 days of recovery, in good clinical condition and in therapy with prednisone, 25 mg daily, and with ebastine, 40 mg daily. Seven days after discharge, serum tryptase was retested, evidencing no significative difference to the level found at the symptom onset (7.4 µg/L). In the next 5 months, the patient experienced two more episodes of urticaria, followed by chest pain after a few days. Both episodes occurred after corticosteroids suspension, with elevation of troponin-I levels (186.6 ng/L in the first one; 191.7 ng/L in the second one) and with ECG displaying ST-segment depression, clinically configurating angina pectoris due to a recurrence of Kounis syndrome in a setting of urticaria. Urticaria was defined as chronic and spontaneous (CSU), considering that it had lasted more than 6 weeks and no triggering factors had been found. In the two mentioned cases, the patient was recovered in the Cardiology Unit where cardiac symptoms promptly responded to sublingual nitroglycerine in both circumstances. The patient was dismissed 8 days after the first hospitalization and 9 days after the second one, and in both cases corticosteroid therapy (prednisone at 25 mg daily) was reinstated for both Kounis syndrome and urticaria exacerbation. Twenty days after the patient’s dischargement, since the patient had shown no control of urticaria with high-dose antihistamines and was not able to suspend oral corticosteroids, our Allergy Unit decided to start omalizumab 150 mg, 2 vials every 4 weeks. In the next 2 months, oral corticosteroids were gradually tapered until suspension without any cutaneous or cardiac symptoms recurrence. After 6 months of omalizumab treatment, anti-IgE therapy was suspended due to Agenzia Italiana del Farmaco restrictions, mandatory at the time. Two months after omalizumab suspension, the patient was admitted to the Emergency Unit of our hospital complaining of the recurrence of urticaria and multiple daily episodes of chest pain. Laboratory examinations showed serum tryptase in a normal range (5.2 µg/L), whereas ECG displayed ST-segment depression, and troponin-I enzymes were elevated (186.6 ng/L), configurating a relapse of angina pectoris. The patient promptly responded to sublingual nitroglycerine and prednisone, 25 mg daily, was also reinstated. Considering the severity of the situation, Agenzia Italiana del Farmaco allowed us to restart omalizumab treatment 3 days after symptom onset. The patient was discharged in good clinical condition after 11 days of recovery. Prednisone was again tapered until suspension after 2 months, and urticaria and cardiac symptoms were generally well-controlled for 8 months. Nevertheless, during this period, the patient experienced other two mild episodes of urticaria and chest pain, both during infectious diseases: the first one during the course of SARS-CoV-2 infection and the second one during the course of influenza. Cardiac and cutaneous symptoms responded to sublingual nitroglycerine and corticosteroids, home-administered on both occasions.

### 2.2. Literature Review

In our research, we found four cases of recurrent type-III Kounis syndrome.

Ma et al. reported a case of a 54-year-old woman, known to have had three polymer-free sirolimus-eluting stents implanted six months earlier after angina in three major coronary arteries, presented to the emergency department complaining of retrosternal chest pain. After stent implantation, the patient was hospitalized with similar complaints two more times: once for single in-stent restenosis and the other for multiple in-stent restenosis. Upon readmission, emergent coronary angiography revealed that multiple coronary arteries had significant restenosis and occlusion. Thrombus aspiration and percutaneous transluminal coronary angioplasty were successful in all involved coronary arteries (grade III myocardial infarction flow thrombolysis). Subsequently, a positron emission tomography/computed tomography with 18F-fluorodeoxyglucose (18F-FDG PET/CT) was performed in combination with coronary CT to assess the presence of active inflammation. The images demonstrated intense FDG uptake in the corresponding restenosis stent segments. Although 18F-FDG uptake may be partly explained by the relatively recent coronary intervention and subsequent inflammation, the images are very suggestive of active coronary inflammation itself. Indeed, thrombus samples stained with hematoxylin-eosin and Giemsa demonstrated the presence of eosinophils and mast cells. Ultimately, the patient was diagnosed with recurrent Kounis syndrome type III. The patient’s condition remained stable after the administration of additional corticosteroids and antihistamine therapy [21].

Fialho et al. reported a case of a 59-year-old man with recurrent Kounis syndrome. The patient presented typical symptoms of acute coronary syndrome (ACT), which started 12 h after the anti-influenza vaccine administration. However, the patient went to hospital only seven days later with heart failure symptoms due to acute left ventricular dysfunction caused by occlusion of the left anterior descending (LAD) coronary artery and proximal stenosis of the right coronary artery (RCA). The latter was treated with one drug-eluting stent (DES) placement. Four months later, the same patient presented typical angina 20 min after the anti-SARS-CoV-2 vaccine administration. Electrocardiogram showed inferior ST-elevation myocardial infarction, and coronary angiography confirmed right coronary artery stent thrombosis. Both vaccines share polysorbate 80 (PS80), identified as the potential trigger of both events. Considering the reproducibility of the reaction and the temporal association between vaccine administration and coronary events, Kounis syndrome due to a hypersensitivity reaction to PS80 was acknowledged as the most probable diagnosis although systemic symptoms of hypersensitivity reaction did not occur. Skin tests with intravenous amiodarone (containing PS80) were negative. A non-IgE-mediated anaphylactoid reaction to PS80 was identified, and the patient was instructed to avoid drugs containing it [18].

Ferreira et al. reported a case compatible with type III Kounis syndrome since the association of systemic allergic manifestations and evidence of new-onset myocardial ischemia after recent percutaneous coronary intervention (PCI). A 57-year-old man with hypertension, dyslipidemia and no history of allergies presented to the emergency department with acute chest pain of 2 h duration. Electrocardiogram showed ST-segment elevation; therefore, aspirin (already taken before) and clopidogrel were administrated, and primary PCI was performed through the RCA, which was occluded with a significant number of thrombi. Thereafter, a total of three DES were placed with an angiographically successful outcome and a resolution of the ST-segment deviation and chest pain. Then, 19 h after the procedure and 1 h after receiving the maintenance dose of clopidogrel and aspirin, the patient developed a macular, non-pruriginous rash, predominantly on the thorax and abdomen and complained of a low-intensity left-sided chest discomfort with recurrence of the ST-segment elevation on electrocardiogram. A considerable number of thrombi occluded the RCA, which is why another DES was placed after aspiration thrombectomy and clopidogrel was replaced by prasugrel. Eighteen hours after the second intervention, the patient remained clinically stable and asymptomatic, although still with the same rash. Continuous electrocardiogram monitoring revealed a reappearance of the ST-segment deviation in addition to a second-degree atrioventricular block. A proximal in-stent thrombosis occluding the RCA was demonstrated, and subsequently, two additional DES were placed and dual antiplatelet therapy with aspirin and prasugrel was continued. Since a suspicion of type III Kounis syndrome had been raised, oral corticosteroids and oral anti-histamine therapies were prescribed after the third intervention. These therapies were maintained during the next three days, after which the rash subsided and no further dynamic electrocardiographic changes were identified. Thereafter, the patient had an uneventful recovery and continued dual anti-platelet therapy with aspirin and prasugrel without other coronary problems [20].

Finally, Liu et al. reported a case of a 48-year-old man presenting two cases of urticaria associated with chest pain after eating scallions. Coronary angiography showed an occlusion of LAD, where a stent had already been placed, and the condition was resolved by percutaneous transluminal coronary angioplasty in both episodes. After the latest episode, omalizumab treatment of 150 mg every 4 weeks was started, with no further recurrence of chest pain or urticaria [19].

The literature also reports a case of recurrent Kounis syndrome associated with loxoprofene. Masuda et al. described a case of a 52-year-old woman who developed drug-induced ventricular fibrillation (VF) in the absence of obvious allergic symptoms after dental treatment including loxoprofene. The patient had no remarkable medical history, allergies or a family history of sudden cardiac death. Ten months earlier, the patient underwent subcutaneous implantable cardioverter-defibrillator implantation based on the previous diagnosis of VF after dental treatment and administration of loxoprofen before VF onset. The patient underwent a loxoprofen provocation test in the ward with the development of electrocardiogram ST-segment elevation and increased histamine level in a laboratory test. These findings strongly supported the diagnosis of Kounis syndrome without typical allergic symptoms. After discharge, no recurrence occurred following loxoprofen avoidance [4].

Finally, Gunaydin et al. reported a case of a 45-year-old patient who had presented at the emergency room complaining of half an hour’s retrosternal chest pain accompanied by weakness and sweating that had begun after a bee sting. The patient was conscious but pale and sweaty. Blood pressure was 70/50 mm Hg, the pulse was 56 rpm and oxygen saturation was 96%. The patient denied any complaints such as itching or skin problems that could be associated with an allergic reaction. The electrocardiogram showed ST-segment elevation in DII, DIII and aVF. Transthoracic echocardiography showed hypokinesia in the cardiac inferior wall. Kounis syndrome was considered the most suitable diagnosis, so therapy with acetylsalicylic acid 300 mg, epinephrine 1 mg IV and infusion of isotonic solution (NaCl 0.9%) was administered, whereupon blood pressure gradually increased. On physical examination 15 min later, blood pressure was 118/85 mm Hg and chest pain subsided. Laboratory tests conducted upon admission revealed mild leukocytosis (WBC count = 12,380/μL) and eosinophilia (1.2%). The IgE level was elevated (180 g/L). During the 4 days of hospitalization in the coronary intensive care unit, troponin I levels remained stable and chest pain did not recur. The patient was clinically and hemodynamically stable during the hospitalization period and was then discharged from the hospital and prescribed diltiazem 60 mg once daily, isosorbide mononitrate 20 mg once daily and desloratadine 5 mg once daily to be taken orally. The history of the patient revealed that she had been admitted to hospital a year earlier with the same complaints following a bee sting, after which she underwent coronary angiography with a diagnosis of acute myocardial infarction. She was discharged from hospital without any prophylactic therapy [22].

## 3. Discussion

In literature, most cases of recurrent Kounis syndrome seem to accompany type III Kounis syndrome, though the four cases of type III Kounis syndrome we reported are quite different from one another. The case described by Fialho et al. is similar to the case we reported, since in both cases Kounis syndrome was triggered by vaccination. According to the authors, Kounis syndrome was triggered by a non-IgE-mediated reaction to PS-80 [18]. Considering that PS-80 is present in countless drugs and also in food, Kounis syndrome induced by hypersensitivity to PS-80 seems unlikely, because, in such a case, the patient would have probably experienced symptoms on multiple other occasions. The temporal association between vaccination and disease onset leads us to consider vaccination a trigger of the symptom outbreak, as described in many studies for CSU [23,24]. In effect, in the case described by Liu et al., Kounis syndrome was associated with chronic urticaria just like it occurred in our case and two other cases reported in the literature [19,25]. More precisely, in CSU elicitation after vaccination, the activation of mast cell toll-like receptors and complement receptors, and the production of anti-FcεR auto-antibodies induced by the vaccine spike protein action are some of the potential mechanisms proposed [24]. Infectious diseases may also act as triggers for both Kounis syndrome and CSU, as reported in our case and described in the literature [26,27]. In CSU guidelines, when treatment with a high dose of second-generation anti-histamines is not effective, therapy with omalizumab is recommended [28]. In our case, omalizumab therapy appeared to be effective in preventing Kounis syndrome recurrence, as it was also evidenced by Liu et al. [19]. As already anticipated by Kounis NG et al. [29], omalizumab can efficiently control recurrent Kounis syndrome related to CSU. The hypothesis behind omalizumab efficacy in this particular condition might be that recurrent Kounis syndrome and CSU, when related to each other, share the same underlying pathogenetic mechanisms. In conclusion, treatment with omalizumab might represent the best therapeutic option for recurrent Kounis syndrome associated with CSU resistant to a high dose of second-generation antihistamines. It is important to underline that in our case report, though the patient achieved good general control of both skin and cardiac symptoms with omalizumab, she still experienced several episodes of Kounis syndrome exacerbation with concomitant infectious disease. In these cases, immediately starting oral corticosteroids and sublingual nitroglycerine as soon as symptoms of a probable infection appear could be an appropriate therapeutic strategy.

The cases reported by Masuda et al. and Gunaydin et al. seem more compatible with isolated episodes of cardiac anaphylaxis than the cases of recurrent Kounis syndrome, considering that NSAIDs are well known to cause Kounis syndrome via hypersensitivity mechanism, which can be IgE-mediated or not, and bee sting is a very common cause of anaphylaxis [4,22,30]. In the literature, cases of Kounis syndrome related to drugs different from NSAIDs are also reported. Common drugs such as losartan and corticosteroids were found as triggers for Kounis syndrome. Losartan is an angiotensin II receptor antagonist frequently prescribed for hypertension treatment. Despite its widespread use, allergic reactions have rarely been reported [31] and cardiovascular events with involvement of the coronary arteries are even more uncommon. However, the association between losartan use and frequently repeated angina pectoris attacks and coronary artery spasms progressing to acute myocardial infarction with electrocardiographic abnormalities and an increase in troponin assay on blood sampling resembling Kounis syndrome have been published [32,33]. Corticosteroids are anti-inflammatory drugs widely used for the treatment of allergic, cutaneous, respiratory, rheumatic, kidney and other diseases as well as a therapy to prevent the rejection of transplants. These drugs are also used in the treatment of forms of refractory angina associated with vasospasm, especially when the patient suffers from different allergic conditions, such as bronchial asthma [34]. However, corticosteroids may sometimes cause allergic reactions up to and including anaphylaxis. A young patient with coronary arteries without structural anomalies developed acute myocardial infarction resembling Kounis syndrome after he received prednisolone used as a treatment for anaphylaxis due to hymenoptera venom after a wasp sting [35].

In the case reported by Liu et al., the pathogenetic role of scallops is questionable because there is no relation between food allergy and CSU, and there is no evidence in the report of skin testing or challenge testing. It is more likely that the scallop worked as a triggering agent without an IgE-mediated hypersensitivity [19].

Finally, it is interesting to point out that Ma et al. used 18F-FDG PET/CT to confirm Kounis syndrome diagnosis [21]. Since Kounis syndrome is a condition frequently misdiagnosed or underdiagnosed, this technology might help in cases where the diagnosis is uncertain.

## 4. Conclusions

Kounis syndrome is a condition where inflammatory cells cause an acute coronary syndrome, mostly via mast cell activation. Its incidence is difficult to assess because Kounis syndrome is frequently misdiagnosed. Kounis syndrome is nearly always an isolated condition; therefore, although Kounis syndrome cannot be considered an uncommon disease, recurrent Kounis syndrome is very rare, with few cases reported in the literature. In most of these cases, recurrent Kounis syndrome is related to type III Kounis syndrome, but it could also be triggered by vaccination or infections and associated with CSU. Kounis syndrome is an unpredictable disease. Serum tryptase level should always be checked in suspicion of Kounis syndrome to define if the origin of the disease is IgE-mediated or not. If Kounis syndrome is IgE-mediated, the strategy to prevent a recurrence is to find and avoid the culprit. If the pathogenesis of the syndrome is not IgE-mediated, the patient should be provided with an effective therapy (anti-histamines, corticosteroids, sublingual nytroglicerine, etc.) to promptly treat an exacerbation of the disease. When the first-line therapy, represented by second-generation anti-histamines and corticosteroids, is not effective or corticosteroid-related side effects occur, omalizumab should be considered to achieve control of the disease and to spare corticosteroids. New methods such as 18F-FDG PET/CT should be considered to help diagnosis in uncertain cases.

## 5. Future Directions

Recurrent Kounis syndrome is a very rare condition, but its low prevalence might be also due to underdiagnosis because of the poor knowledge of the topic. It would be important to raise awareness of recurrent Kounis syndrome among all clinicians, especially among cardiologists and doctors handling emergency units, to reduce the frequency of misdiagnosis of “idiopathic coronaropathy”, promote the use of appropriate diagnostic tests such as serum tryptase, avoid ineffective treatment such as multiple stenting and promptly establish appropriate therapy. This goal might be achieved by creating awareness campaigns at medical conventions or via the World Wide Web, using social media or e-mails.

## References

[1] Kounis N.G. (2016). Kounis Syndrome: An Update on Epidemiology, Pathogenesis, Diagnosis and Therapeutic Management. Clin. Chem. Lab. Med..

[2] Goto M., Matsuzaki M., Fuchinoue A., Urabe N., Kawagoe N., Takemoto I., Tanaka H., Watanabe T., Miyazaki T., Takeuchi M. (2012). Chronic Atherosclerotic Mesenteric Ischemia That Started to Develop Symptoms Just after Anaphylaxis. Case Rep. Gastroenterol..

[3] González-De-Olano D., Álvarez-Twose I., Matito A., Sánchez-Muñoz L., Kounis N.G., Escribano L. (2011). Mast Cell Activation Disorders Presenting with Cerebral Vasospasm-Related Symptoms: A “Kounis-like” Syndrome?. Int. J. Cardiol..

[4] Masuda M., Fujimoto W., Yamashita S., Takemoto M., Kuroda K., Imanishi J., Iwasaki M., Todoroki T., Okuda M., Hayashi T. (2022). Recurrent Cardiac Arrests Caused by Kounis Syndrome without Typical Allergic Symptoms. J. Cardiol. Cases.

[5] Helbling A., Hurni T., Mueller U.R., Pichler W.J. (2004). Incidence of Anaphylaxis with Circulatory Symptoms: A Study over a 3-Year Period Comprising 940,000 Inhabitants of the Swiss Canton Bern. Clin. Exp. Allergy.

[6] Kounis N.G. (2013). Coronary Hypersensitivity Disorder: The Kounis Syndrome. Clin. Ther..

[7] Hasegawa S., Tashiro N., Matsubara T., Furukawa S., Ra C. (2001). A Comparison of FcepsilonRI-Mediated RANTES Release from Human Platelets between Allergic Patients and Healthy Individuals. Int. Arch. Allergy Immunol..

[8] Mast Cell Mediators Stimulate Synthesis of Arachidonic Acid Metabolites in Macrophages—PubMed. https://pubmed.ncbi.nlm.nih.gov/2495326/.

[9] Mejía-Rentería H.D., Viana-Tejedor A., Sánchez-Enrique C., Nombela L., Herrera J.P., Ruiz-Mateos B., Núñez-Gil I.J., Vivas D., Fernández-Ortiz A., MacAya C. (2014). Kounis Syndrome after Ingestion of Undercooked Fish: New Role of Intracoronary Imaging Techniques. Int. J. Cardiol..

[10] Wickman M. (2005). When Allergies Complicate Allergies. Allergy.

[11] Carl-Mcgrath S., Grntzdrffer I., Lendeckel U., Ebert M.P., Rcken C. (2009). Angiotensin II-Generating Enzymes, Angiotensin-Converting Enzyme (ACE) and Mast Cell Chymase (CMA1), in Gastric Inflammation May Be Regulated by H. Pylori and Associated Cytokines. Pathology.

[12] Riccioni G., Zanasi A., Vitulano N., Mancini B., D’Orazio N. (2009). Leukotrienes in Atherosclerosis: New Target Insights and Future Therapy Perspectives. Mediators Inflamm..

[13] Arshad M., Vijay V., Floyd B.C., Marks B., Sarabu M.R., Wolin M.S., Gupte S.A. (2006). Thromboxane Receptor Stimulation Suppresses Guanylate Cyclase-Mediated Relaxation of Radial Arteries. Ann. Thorac. Surg..

[14] Castells M.C., Irani A.M., Schwartz L.B. (1987). Evaluation of Human Peripheral Blood Leukocytes for Mast Cell Tryptase. J. Immunol..

[15] Kounis N.G. (2007). Serum Tryptase Levels and Kounis Syndrome. Int. J. Cardiol..

[16] Goto K., Kasama S., Sato M., Kurabayashi M. (2016). Myocardial Scintigraphic Evidence of Kounis Syndrome: What Is the Aetiology of Acute Coronary Syndrome?. Eur. Heart J..

[17] Okur A., Kantarci M., Karaca L., Ogul H., Aköz A., Kizrak Y., Aslan S., Pirimoglu B., Aksakal E., Emet M. (2015). The Utility of Cardiac Magnetic Resonance Imaging in Kounis Syndrome. Postępy Kardiol. Interw. Adv. Interv. Cardiol..

[18] Fialho I., Mateus C., Martins-dos-Santos G., Pita J., Cabanelas N., Baptista S.B., Roque D. (2022). Recurrent Kounis Syndrome—A Life-Threatening Event after COVID-19 Vaccine Administration. J. Cardiol. Cases.

[19] Liu Y., Lu C., Guo Q. (2020). Recurrent Type III Kounis Syndrome: Will Anti-Immunoglobulin E Drug Be Another Option?. Can. J. Cardiol..

[20] Ferreira R.M., Villela P.B., Almeida J.C.G., Sampaio P.P.N., Albuquerque F.N., Pinheiro F.M.C., França Filho W., Salles J.A.B.e., Mansur Filho J. (2018). Allergic Recurrent Coronary Stent Thrombosis: A Mini-Review of Kounis Syndrome. Cardiovasc. Revasc. Med..

[21] Ma C., Wang X., Cheng Z. (2022). Recurrent Type III Kounis Syndrome: Coronary Artery Inflammation Assessed by 18F-Fluorodeoxyglucose Positron Emission Tomography/Computer Tomography. Eur. Heart J. Cardiovasc. Imaging.

[22] Günaydin Z.Y., Bektaş O., Akgedik R., Kaya A., Acar T. (2014). Recurrent Kounis Syndrome. How Should Be the Long-Term Treatment of Kounis Syndrome?. Int. J. Cardiol..

[23] Magen E., Yakov A., Green I., Israel A., Vinker S., Merzon E. (2022). Chronic Spontaneous Urticaria after BNT162b2 MRNA (Pfizer-BioNTech) Vaccination against SARS-CoV-2. Allergy Asthma Proc..

[24] de Montjoye L., Herman A., Baeck M. (2022). Chronic Spontaneous Urticaria Following COVID-19 Vaccination. JAAD Case Rep..

[25] Erxun K., Wei L., Shuying Q. (2016). Kounis Syndrome Caused by Chronic Autoimmune Urticaria: A Case Report. J. Emerg. Med..

[26] Kounis N.G., Gogos C., de Gregorio C., Hung M.Y., Kounis S.N., Tsounis E.P., Assimakopoulos S.F., Pourmasumi S., Mplani V., Servos G. (2024). “When”, “Where”, and “How” of SARS-CoV-2 Infection Affects the Human Cardiovascular System: A Narrative Review. Balkan Med. J..

[27] Imbalzano E., Casciaro M., Quartuccio S., Minciullo P.L., Cascio A., Calapai G., Gangemi S. (2016). Association between Urticaria and Virus Infections: A Systematic Review. Allergy Asthma Proc..

[28] Zuberbier T., Abdul Latiff A.H., Abuzakouk M., Aquilina S., Asero R., Baker D., Ballmer-Weber B., Bangert C., Ben-Shoshan M., Bernstein J.A. (2022). The International EAACI/GA^2^LEN/EuroGuiDerm/APAAACI Guideline for the Definition, Classification, Diagnosis, and Management of Urticaria. Allergy Eur. J. Allergy Clin. Immunol..

[29] Kounis N.G., Koniari I., Tsigkas G., Davlouros P. (2020). Humanized Monoclonal Antibodies Against IgE Antibodies as Therapy for IgE-Mediated Coronary Syndromes: Are We There Yet?. Can. J. Cardiol..

[30] El Hangouche A.J., Lamliki O., Oukerraj L., Dakka T., Doghmi N., Zarzur J., Cherti M. (2018). Kounis Syndrome Induced by Oral Intake of Aspirin: Case Report and Literature Review. Pan Afr. Med. J..

[31] Losartan Associated Anaphylaxis and Angioneurotic Oedema—PubMed. https://pubmed.ncbi.nlm.nih.gov/20726206/.

[32] Losartan and Angina Pectoris—PubMed. https://pubmed.ncbi.nlm.nih.gov/8605440/.

[33] Josefsson J., Fröbert O. (2012). Losartan-Induced Coronary Artery Spasm. BMJ Case Rep..

[34] Takagi S., Goto Y., Hirose E., Terashima M., Sakuragi S., Suzuki S., Tsutsumi Y., Miyazaki S., Nonogi H. (2004). Successful Treatment of Refractory Vasospastic Angina with Corticosteroids: Coronary Arterial Hyperactivity Caused by Local Inflammation?. Circ. J..

[35] Arslan Z., Iyisoy A., Tavlasoglu M. (2013). Acute Myocardial Infarction after Prednisolone Administration for the Treatment of Anaphylaxis Caused by a Wasp Sting. Cardiovasc. J. Afr..

